# The Theranostic Optimization of PSMA-GCK01 Does Not Compromise the Imaging Characteristics of [^99m^Tc]Tc-PSMA-GCK01 Compared to Dedicated Diagnostic [^99m^Tc]Tc-EDDA/HYNIC-iPSMA in Prostate Cancer

**DOI:** 10.1007/s11307-023-01881-y

**Published:** 2023-12-08

**Authors:** Eduards Mamlins, Lara Scharbert, Jens Cardinale, Maria Krotov, Erik Winter, Hendrik Rathke, Birgit Strodel, Alfred O. Ankrah, Mike Sathekge, Uwe Haberkorn, Clemens Kratochwil, Frederik L. Giesel

**Affiliations:** 1 Department of Nuclear Medicine, Medical Faculty, University Hospital Dusseldorf, Heinrich-Heine-University Dusseldorf, Moorenstrasse 5, 40225 Dusseldorf, Germany; 2 Institute of Biological Information Processing: Structural Biochemistry (IBI-7), Forschungszentrum Juelich GmbH, Juelich, Germany; 3 Institute of Theoretical and Computational Chemistry, Heinrich-Heine-University Dusseldorf, Dusseldorf, Germany; 4 Department of Nuclear Medicine, University Hospital Heidelberg, Heidelberg, Germany; 5 Department of Nuclear Medicine, University Hospital of Bern - Inselspital, Bern, Switzerland; 6 Korle Bu Teaching Hospital, Accra, Ghana; 7 Department of Nuclear Medicine, University of Pretoria and Steve Biko Academic Hospital, Pretoria, South Africa; 8 Clinical Cooperation Unit Nuclear Medicine, DKFZ, Heidelberg, Germany; 9 Translational Lung Research Center Heidelberg (TLRC), German Center for Lung Research (DZL), Heidelberg, Germany

**Keywords:** PSMA-GCK01, Technetium-99 m, SPECT, Theranostic, Prostate cancer

## Abstract

**Purpose:**

Radiolabeled PSMA-ligands play a major role in today’s nuclear medicine. Since approval of [^177^Lu]Lu-PSMA-617 for therapy of metastatic prostate cancer, availability of ^177^Lu became bottleneck of supply due to the high demand. Recently, a theranostic PSMA-ligand, PSMA-GCK01, was developed which can be labeled either diagnostically with ^99m^Tc or therapeutically with ^188^Re with both nuclides available from well-known generator systems. This novel tracer might aid to overcome aforementioned supply limitations. In this investigation, the biodistribution and general imaging characteristics of [^99m^Tc]Tc-PSMA-GCK01 were compared with the diagnostic reference compound [^99m^Tc]Tc-EDDA/HYNIC-iPSMA in patients with advanced stage prostate cancer. In addition, the binding of both ligands to PSMA was analyzed at the molecular level using molecular docking.

**Procedures:**

Two cohorts (*n* = 19 vs. *n* = 21) of patients with metastatic castration-resistant prostate cancer matched for age, tumor stage, and Gleason score underwent a planar gamma camera imaging with [^99m^Tc]Tc-EDDA/HYNIC-iPSMA or [^99m^Tc]Tc-PSMA-GCK01 prior to PSMA-ligand therapy for PSMA-phenotyping. The imaging data were retrospective analyzed for salivary gland, kidney, liver, soft tissue, and tumor uptake on a semi-automated ROI-analysis using HERMES Medical Solutions AB (HMS, Sweden).

**Results:**

The data sets were semi-automated quantified on a ROI-based analysis. The tumor-to-background presented equal results of [^99m^Tc]Tc-PSMA-GCK01 compared to [^99m^Tc]Tc-EDDA/HYNIC-iPSMA. The physiological PSMA-positive organs like salivary gland presented also equal uptake in counts/MBq (salivary gland median 9.48 [^99m^Tc]Tc-PSMA-GCK01 vs. median 9.11 [^99m^Tc]Tc-EDDA/HYNIC-iPSMA), while liver-to-kidney ratio presented a slight shift to the liver parenchyma using [^99m^Tc]Tc-PSMA-GCK01 (0.83) compared to [^99m^Tc]Tc-EDDA/HYNIC-iPSMA (0.55) with no statistical significance. This is in agreement with the results from the docking study revealing only a minor difference in the docking scores for both ligands.

**Conclusions:**

The novel theranostic tracer [^99m^Tc]Tc/[^188^Re]Re-PSMA-GCK01 demonstrates comparable general imaging characteristic with the reference compound [^99m^Tc]Tc-EDDA/HYNIC-iPSMA. These results pave the way for the PSMA-targeting imaging and theranostic agents for a broader, rather low-cost, generator applied radio-ligand therapy utilization.

## Introduction

The development of prostate-specific membrane antigen (PSMA)-inhibitor tracers has revolutionized the imaging of prostate cancer. PSMA PET tracers [^68^Ga]Ga-PSMA-11, [^18^F]PSMA-1007, and [^18^F]DCFPyL demonstrate high sensitivity of approx. 50% at PSA values as low as 0.2–0.5 ng/mL and reach their plateau sensitivity of approx. 90% from PSA > 1 ng/mL [1–3]. In contrast, ^99m^Tc labeled SPECT imaging PSMA agents reach their highest sensitivity not before PSA 2–5 ng/mL [4–6]. Consequently, PSMA-PET is considered the standard of reference for primary staging of high-risk prostate cancer (PC) and biochemical recurrence. However, for response assessment of advanced stage patients [7] or tailoring metastatic castration-resistant prostate cancer (mCRPC) patients with or without tumor PSMA-expression to PSMA-targeted radioligand therapy (RLT) highest sensitivity is not that pivotal and cost-effectiveness and broad availability are more important [8]. It is worth mentioning in this context that a randomized phase 3 clinical study of ^177^Lu-dotatate for midgut neuroendocrine tumors (NETTER-1 trial) used planar somatostatin receptor scintigraphy for the confirmation of somatostatin receptor positive tumor lesions [9]. Currently, most clinical experience in context of PSMA targeted scintigraphies is available for the ^99m^Tc-tracers [^99m^Tc]Tc-MIP1404 [10], [^99m^Tc]Tc-EDDA/HYNIC-iPSMA [11], and [^99m^Tc]Tc-PSMA-I&S [12].

Recently, a novel theranostic PSMA-ligand GCK01, that can be labeled with diagnostic ^99m^Tc and therapeutic ^188^Re, has been introduced [13]. The generator-based beta-emitter ^188^Re may aid to overcome supply limitations of ^177^Lu and decrease the production costs for PSMA-RLT in comparison to ^177^Lu. In contrast to the previous Tc-labeled ligands, GCK01 had to be refined with regard to labeling yield under more challenging rhenium conditions as well as in terms of tracer uptake in the dose-limiting organs. In addition, the *in vivo* stability has been improved, which represents a pivotal cornerstone for the therapeutic approach.

The aim of this work is to evaluate whether the optimizations of PSMA-GCK01 for the therapeutic application might have introduced an unfavorable trade-off regarding biodistribution in normal organs and general imaging characteristics.

## Materials and Methods

### Patient Population

We retrospectively included two cohorts of 21 consecutive patients with mCRPC who were considered to obtain systemic therapy with [^177^Lu]Lu-PSMA-617 and underwent whole body scans with [^99m^Tc]Tc-EDDA/HYNIC-iPSMA or [^99m^Tc]Tc-PSMA-GCK01 in the Department of Nuclear Medicine of University Hospital Heidelberg between 01/2021 and 02/2022. Two subjects from [^99m^Tc]Tc-EDDA/HYNIC-iPSMA cohort were censored due to ineligibility for further quantitative assessment because of improper image acquisition with motion artifacts. Informed consent was obtained from all the patients. The exams were conducted according to national regulatory on the basis of compassionate use, when a PSMA-PET scan was not available within reasonable time and the patient had urgently to be evaluated regarding PSMA therapy. Prospective clinical trial registration is not required for compassionate care that is performed under individual medical indication. The Ethical Committee of the University Hospital Heidelberg approved the retrospective Evaluation (permission S-732-18).

### Radiopharmaceuticals and Image Acquisition

[^99m^Tc]Tc-EDDA/HYNIC-iPSMA was produced from commercial kits according manufacturer instructions and [^99m^Tc]Tc-PSMA-GCK01 was synthesized as described previously [13]. Both tracers were injected via an intravenous bolus ([^99m^Tc]Tc-EDDA/HYNIC-iPSMA 669 ± 94 MBq; [^99m^Tc]Tc-GCK01 705 ± 30 MBq). The acquisition of planar whole body images (skull to toes) started for both tracers 120–180 min after the injection on a Siemens gamma camera system (Symbia, Siemens Healthineers) with low-energy high-resolution collimation, a 128 × 128 matrix and 15 cm/min.

### Image Analysis

Round regions of interest (ROIs) (1.6 cm^2^) were placed in the areas with physiological tracer uptake (parotid gland (Gl. parotis) and kidneys pairwise, liver, and in the right thigh as a background) in conjugate view of planar anterior and posterior whole-body scans of the respective subjects and the geometric mean was calculated (HERMES Medical Imaging Suite v6.1, HERMES Medical Solutions AB, Strandbergsgatan 16, 112 51 Stockholm, Sweden). In addition to the measurement of physiological biodistribution in the healthy organs, three random metastases were visually identified and quantified in the same way.

### Statistical Analysis

We performed the analysis of tracer uptake (measured counts divided by the injected activity in MBq), tumor-to-background, as well as liver-to-kidney ratios with descriptive statistics and box-plot analysis using SigmaPlot version 14.0 (Systat Software, Inc., San Jose, CA, USA).

### Molecular Docking

The crystal structure of PSMA was retrieved from the Protein Data Bank (PDB #5O5T) and processed, i.e., hydrogen atoms were added to the protein, ligand, and water molecules, using the protein preparation wizard of Maestro, included in the Schrödinger 2022–3 package.

To simulate the environment of the binding site as realistically as possible, we kept the ions and all water molecules located in the buried part of the binding pocket. The compounds [^99m^Tc]Tc-PSMA-GCK01 and [^99m^Tc]Tc-EDDA/HYNIC-iPSMA were drawn using Marvin Sketch version 21.9 (https://chemaxon.com/marvin) and exported in 3D to pdb format. The following preparation and docking steps were performed with the software of the ADFR suite version 1.0 (https://ccsb.scripps.edu/adfr/): First, Gasteiger charges were added to the protein, including the ions Zn^2+^, Ca^2+^, and Cl^−^ and the water molecules, using the prepare_receptor script. Then the docking grid was created with AGFR using the co-crystallized ligand 9OT to determine the size and position of the grid box. The prepare_ligand script from the ADFR suite was used to add Gasteiger charges and to define rotatable bonds of the compounds [^99m^Tc]Tc-PSMA-GCK01 and [^99m^Tc]Tc-EDDA/HYNIC-iPSMA. Since Tc and Re are unfamiliar elements for Autodock, we changed the atom type Tc to Mn in the pdbqt files without affecting any atomic coordinates and assigned a charge of + 2 to Mn. Finally, we performed the docking run with 120 searches and a maximum number of 5 million evaluations per search using ADFR.

## Results

Apart from the data of the two [^99m^Tc]Tc-EDDA/HYNIC-iPSMA patients that had to be removed, all other data sets were analyzed the way described above. Our subjects were matched in regard to age ([^99m^Tc]Tc-EDDA/HYNIC-iPSMA 71 ± 10 years; range 55–94 years or [^99m^Tc]Tc-PSMA-GCK01 73 ± 8 years; range 59–84 years), tumor stage, and total Gleason score so that we assume here a comparability of both cohorts (Table 1).

**Table 1:** Patient characteristics of cohort A ([^99m^Tc]Tc-PSMA-GCK01) and cohort B ([^99m^Tc]Tc-EDDA/HYNIC-iPSMA)

	Cohort A	Cohort B
Parameters	[^99m^Tc]Tc-PSMA-GCK01	[^99m^Tc]Tc-EDDA/HYNIC-iPSMA
Age (years)		
Mean ± SD	73 ± 8	71 ± 10
Max	84	94
Min	59	55
PSA (ng/mL)		
Mean ± SD	276.1 ± 613.3	279.2 ± 387.2
Max	2866.8	1399.0
Min	0.1	1.7
Gleason score		
Median (range)	7 (7–10)	9 (6–10)
LDH (U/L)		
Mean ± SD	385.2 ± 195.6	349.8 ± 231.3
Max	957.0	972.0
Min	192.0	185.0
AP (U/L)		
Mean ± SD	267.4 ± 370.5	399.0 ± 487.4
Max	1500.0	1799.0
Min	41.0	31.0
NAAD^a^		
None	1	1
1 cycle	10	6
2 cycle	9	11
Taxane		
None	3	4
1 cycle	6	6
2 cycle	11	8
PSMA positive tumor burden		
Low (< 10 lesions)	8	4
Medium (10–100 lesions)	4	6
High (> 100 lesions or bmc^b^)	9	9

^a^Neoadjuvant androgen deprivation therapy

^b^Bone marrow carcinosis

**Fig. 1:**
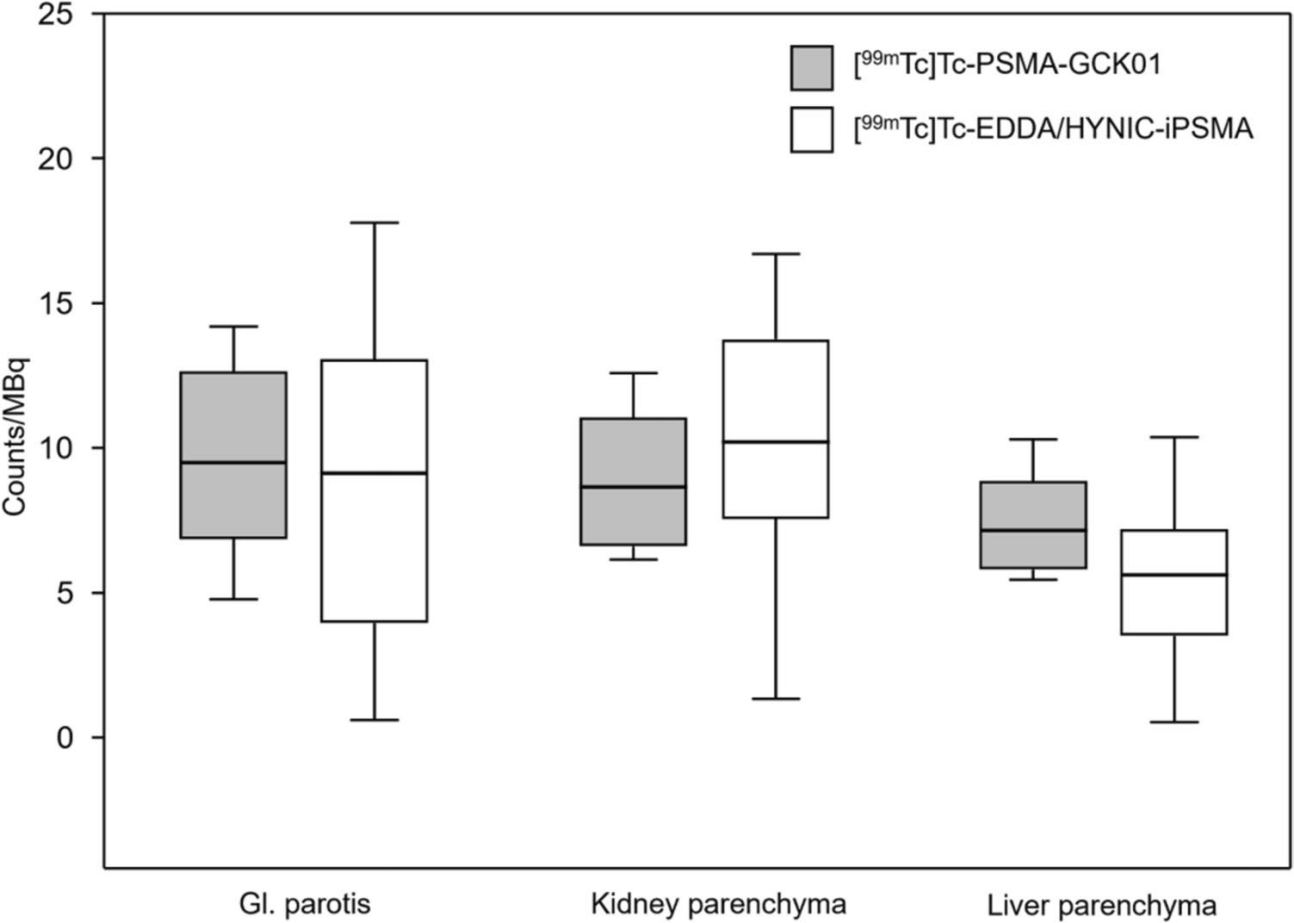
Comparison of physiological biodistribution of [^99m^Tc]Tc-PSMA-GCK01 and [^99m^Tc]Tc-EDDA/HYNIC-iPSMA. Box plots show 5th/95th IQR

Both PSMA ligands demonstrated the highest physiological uptake in counts/MBq in the salivary glands (median 9.48 [^99m^Tc]Tc-PSMA-GCK01 vs. median 9.11 [^99m^Tc]Tc-EDDA/HYNIC-iPSMA), followed by kidneys (median 8.64 [^99m^Tc]Tc-PSMA-GCK01 vs. median 10.20 [^99m^Tc]Tc-EDDA/HYNIC-iPSMA), and the liver (median 7.14 [^99m^Tc]Tc-PSMA-GCK01 vs. median 5.61 [^99m^Tc]Tc-EDDA/HYNIC-iPSMA) (Fig. 1).

**Fig. 2:**
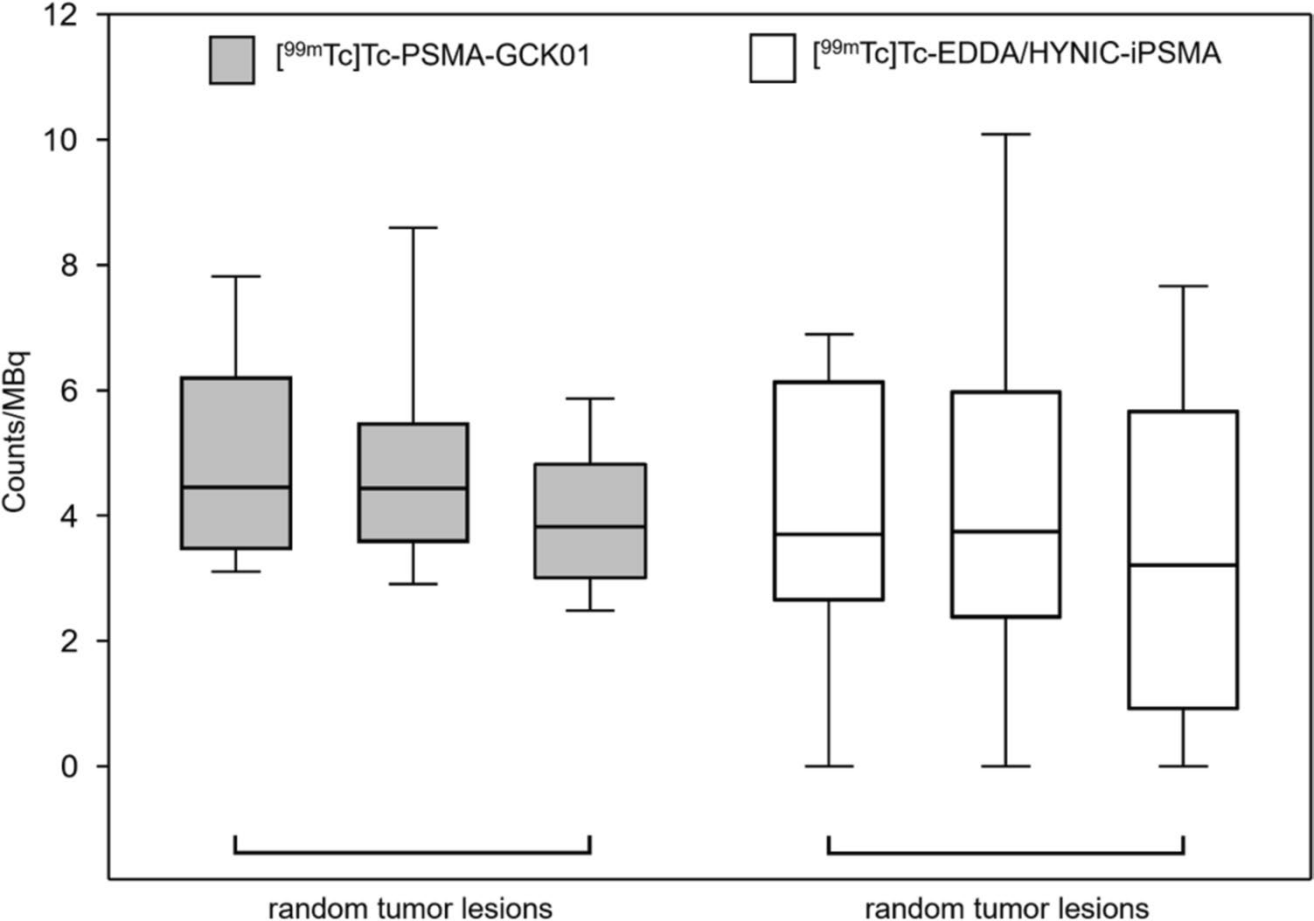
Comparison of [^99m^Tc]Tc-PSMA-GCK01 and [^99m^Tc]Tc-EDDA/HYNIC-iPSMA uptake in three random tumor lesions. Box plots show 5th/95th IQR

**Fig. 3:**
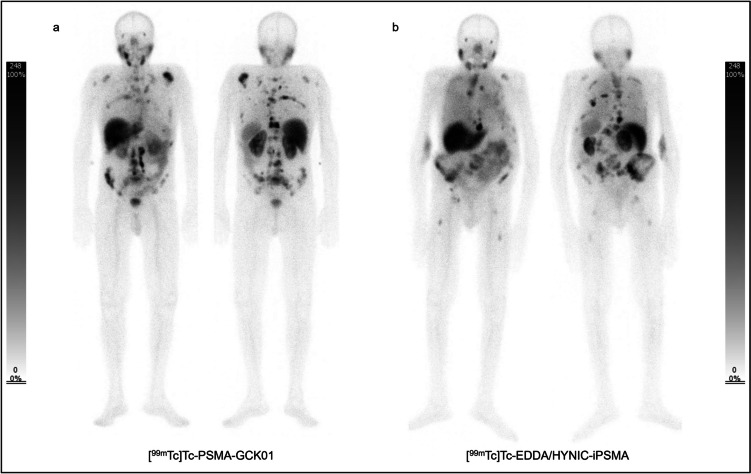
Comparison of **a** [^99m^Tc]Tc-PSMA-GCK01 and **b** [^99m^Tc]Tc-EDDA/HYNIC-iPSMA uptake in whole body projections in patients with high tumor mass. The uptake in metastasis of PSMA-GCK01 is not superior to that of its counterpart by less uptake in kidneys

The uptake was equal in the salivary glands (see above) and in background (counts/MBq median 0.47 [^99m^Tc]Tc-PSMA-GCK01 vs. median 0.44 [^99m^Tc]Tc-EDDA/HYNIC-iPSMA). But the liver-to-kidney ratio presented a slight shift to the liver parenchyma for [^99m^Tc]Tc-PSMA-GCK01 (0.83) compared to [^99m^Tc]Tc-EDDA/HYNIC-iPSMA (0.55), however, with no statistical significance. The uptake in tumor lesions as well as the tumor-to-background ratio presented comparable results for [^99m^Tc]Tc-PSMA-GCK01 and [^99m^Tc]Tc-EDDA/HYNIC-iPSMA, however, with a slightly higher uptake for [^99m^Tc]Tc-PSMA-GCK01 ([[^99m^Tc]Tc-PSMA-GCK01 uptake in tumor lesions in counts/MBq for tumor lesion (TL) I: 4.45; TL II: 4.43; TL III: 3.82 with appropriate tumor-to-background ratio of TL I 9.47; TL II 9.43; TL III 8.12]; [^99m^Tc]Tc-EDDA/HYNIC-iPSMA uptake in tumor lesions in counts/MBq for TL I: 3.69; TL II: 3.74; TL III: 3.20 with appropriate tumor-to-background ratio of TL I 8.36; TL II 8.47; TL III 7.26]) (Figs. 2 and 3).

### Molecular Docking Studies

**Fig. 4:**
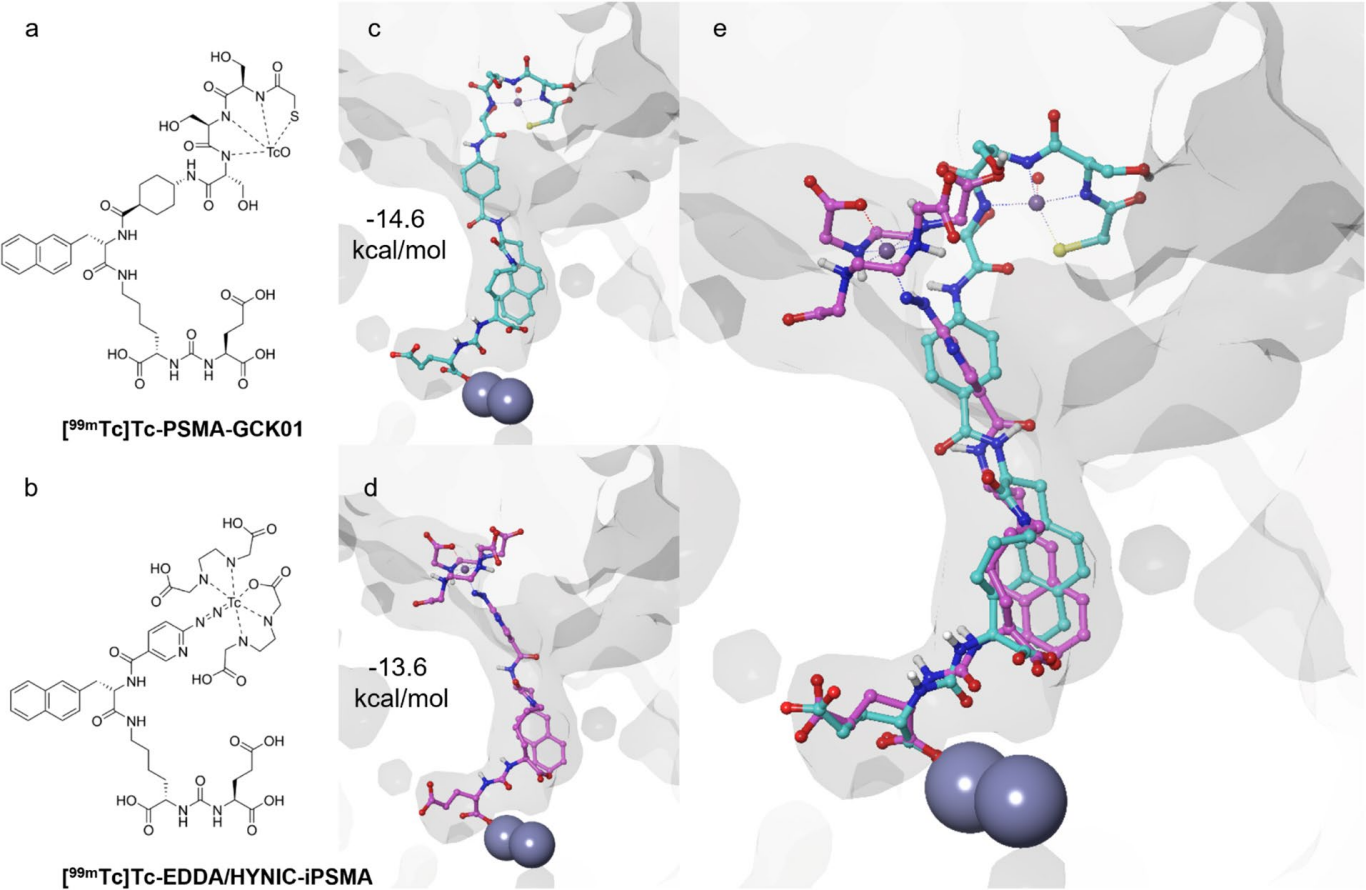
Results from the docking study of [^99m^Tc]Tc-PSMA-GCK01 and [^99m^Tc]Tc-EDDA/HYNIC-iPSMA with the PSMA protein; **a** chemical structure of [^99m^Tc]Tc-PSMA-GCK01 and **b** chemical structure of [^99m^Tc]Tc-EDDA/HYNIC-iPSMA; **c** top-ranked docking pose and score of [^99m^Tc]Tc-PSMA-GCK01 and **d** top-ranked docking pose and score of [^99m^Tc]Tc-EDDA/HYNIC-iPSMA; and **e** overlay of both top-ranked docking poses in PSMA

In order to examine the binding of both ligands in the PSMA receptor pocket, we conducted molecular docking studies. As a measure for the binding affinity, docking scores of − 14.6 kcal/mol for [^99m^Tc]Tc-PSMA-GCK01 and − 13.6 kcal/mol for [^99m^Tc]Tc-EDDA/HYNIC-iPSMA were obtained for the top-ranked conformations (Fig. 4): Both ligands are deeply embedded in the binding pocket, with the urea group interacting with the zinc(II) active site and the glutamate moiety pointing toward the glutamate pocket, while the 2-naphtylalanine occupies a lipophilic cavity and the chelators stick out of the entrance funnel (Fig. 4).

## Discussion

Prostate cancer has an increasing incidence in the world so that there is a need of cost-effective PSMA imaging and therapy [4, 14]. Besides PSMA PET tracers, several [^99m^Tc]Tc-labeled PSMA ligands have been developed in recent years [15]. Contrary to PET, gamma scanners are broadly available throughout the world suggesting the usefulness of compatible radioligands [6, 16].

**Fig. 5:**
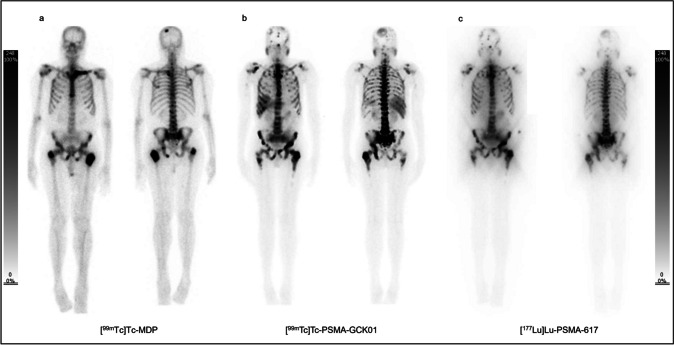
Eighty-one-year-old male patient. This presents an extended prostate cancer undergoing a phenotyping for specific radio-ligand therapy with PSMA. **a** [^99m^Tc]Tc-MDP uptake in whole body projections, **b** [^99m^Tc]Tc-PSMA-GCK01 uptake in whole body projections, and **c** [^177^Lu]Lu-PSMA-617 uptake in whole body projections. [^99m^Tc]Tc-PSMA-GCK01 reveals more lesions compared with [^99m^Tc]Tc-MDP. [^99m^Tc]Tc-PSMA-GCK01 uptake correlates very well with therapeutic biodistribution

The first human data with [^99m^Tc]Tc-MIP-1404 and [^99m^Tc]Tc-MIP-1405 showed very promising results [6, 17]. In 2017, Ferro-Flores et al. introduced [^99m^Tc]Tc-EDDA/HYNIC-iPSMA which offers a rapid blood clearance, the ability to detect prostate cancer, as well as its metastases, and has mainly kidney elimination with less liver uptake than [^99m^Tc]Tc-MIP-1404 [18]. Further investigations with [^99m^Tc]Tc-EDDA/HYNIC-iPSMA demonstrated high detection rates for PSMA positive lesions, especially in the setting of biochemically recurrence of prostate cancer but a lower sensitivity compared with PET PSMA tracer [4, 19]. The main advantage of the new ^99m^Tc-labeled tracer group is the detection of PSMA positive lesions for planning radio-ligand therapy, for instance, with [^177^Lu]Lu-PSMA-617 [8] (Fig. 5). A specific radio-ligand therapy of prostate cancer can only be performed in presence of PSMA-positive phenotype of tumor lesions [20]. The sensitivity of PSMA imaging plays a subordinate role in this context, rather, the diagnostic and therapeutic compounds should be as similar as possible to predict kinetics and uptake of the latter [7, 8].

The novel PSMA ligand [^99m^Tc]Tc-PSMA-GCK01 was optimized to provide a suitable basis for both ^99m^Tc- and ^188^Re-labeling, thus, providing a seamless PSMA-targeted theranostic tandem [13]. In this investigation we have analyzed the biodistribution of the diagnostic [^99m^Tc]Tc-PSMA-GCK01 in organs with physiologic PSMA-uptake and in metastases to assess the appropriateness for imaging, especially in comparison with [^99m^Tc]Tc-EDDA/HYNIC-iPSMA. The ROI-based analysis showed equal tracer uptake in salivary glands and background with [^99m^Tc]Tc-PSMA-GCK01 and [^99m^Tc]Tc-EDDA/HYNIC-iPSMA, respectively.

This observation is also in agreement with the results from our *in silico* docking study, revealing only minimal differences in the conformations of both molecules in the PSMA binding pocket, and, thus, only a minimal difference in the docking score of approx. 1.0 kcal/mol. Considering the high structural similarity of both PSMA ligands, differing only in their chelator moiety and slightly in the linker region, this explains our earlier findings of a slightly higher affinity of [^99m^Tc]Tc-PSMA-GCK01 (Ki = 26 nm) toward PSMA when compared to [^99m^Tc]Tc-EDDA/HYNIC-iPSMA (Ki = 38 nm) [13].

In the practical setting, both tracers had a relevant uptake in the kidneys and the liver. But in the case of [^99m^Tc]Tc-PSMA-GCK01, a minor shift of the uptake to the liver parenchyma was observed. This behavior is particularly interesting because in mice only minimal liver uptake was previously described [13]. Differences in biodistribution between preclinical data and in humans have already been reported for PSMA tracers [21]. Vallabhajosula et al. found a very high liver uptake for [^99m^Tc]Tc-MIP-1404 contrary to preclinical data [17]. [^99m^Tc]Tc-EDDA/HYNIC-iPSMA was optimized for rapid blood clearance; however, more activity is cleared by the kidneys to the bladder [18]. The rapid blood clearance of [^99m^Tc]Tc-EDDA/HYNIC-iPSMA is maintained with [^99m^Tc]Tc-PSMA-GCK01 but the slight predominant hepatobiliary route of excretion of [^99m^Tc]Tc-PSMA-GCK01 might allow a better diagnostic performance of local relapse or perivesical lymph node metastasis [22] since tracer retention in the bladder might complicate the proper discrimination of perivesical lesions. Further, the slight predominant hepatobiliary excretion could be particularly beneficial when the ligand is labeled with ^188^Re in regard to radioligand therapy in terms of nephrotoxicity.

We have also compared the uptake of both tracers in the metastases. The analysis of tumor-to-background ratio presented equal results of [^99m^Tc]Tc-PSMA-GCK01 and [^99m^Tc]Tc-EDDA/HYNIC-iPSMA on the planar whole body projections. Thus, the slight preference for hepatobiliary clearance of [^99m^Tc]Tc-PSMA-GCK01 does not have a negative effect on the imaging properties of tumor lesions and rather offers an optimized biodistribution regarding radioligand therapy, as mentioned above.

The main advantage of [^99m^Tc]Tc-PSMA-GCK01 over [^99m^Tc]Tc-EDDA/HYNIC-iPSMA is its utilization for theranostic purposes by ^188^Re labeling [13, 15]. This ensures the correlation between pre-therapeutic and therapeutic distribution and is expected to have a direct impact on the clinical routine.

Although PSMA PET radioligands and Tc-labeled PSMA SPECT tracers use the same target structure, there are differences in sensitivity [19] and lesion-to-liver ratio. The latter is important in case of eligibility for [^177^Lu]Lu-PSMA therapy and needs to be adjusted if ^99m^Tc labeled tracers are used for the pre-therapeutic diagnostic [8, 23]. The tracer PSMA-GCK01 labeled with ^99m^Tc-/^188^Re-theranostic tandem could solve this problem [13] without negatively affecting the biodistribution and general imaging characteristics compared with [^99m^Tc]Tc-EDDA/HYNIC-iPSMA.

## Conclusions

[^99m^Tc]Tc-PSMA-GCK01 shows a similar uptake (counts/MBq) in normal organs and background, consequently leading to equivalent contrast ratios and general uptake in tumor lesions compared to [^99m^Tc]Tc-EDDA/HYNIC-iPSMA. [^99m^Tc]Tc-PSMA-GCK01 presents a small shift from kidney to liver parenchyma in the clearance route, which is an appreciated characteristic regarding its therapeutic use but not a relevant factor for diagnostics. In sum, its additional appropriateness for use as a therapeutic agent does not compromise the general imaging characteristics of [^99m^Tc]Tc-PSMA-GCK01 compared to the already well-established [^99m^Tc]Tc-EDDA/HYNIC-iPSMA.

## Data Availability

The data used and/or analyzed during the current study are available from the corresponding author on reasonable request.
